# Hybrid control combined with a voluntary biosignal to control a prosthetic hand

**DOI:** 10.1186/s40638-018-0087-5

**Published:** 2018-09-19

**Authors:** Saeed Bahrami Moqadam, Seyed Mohammad Elahi, An Mo, WenZeng Zhang

**Affiliations:** 1 Department of Mechanical Engineering, Tsinghua University, Beijing, China; 2 Department of Nuclear Science Engineering, Shahid Beheshti University, Tehran, Iran

**Keywords:** EMG, PD, Fuzzy logic, Two DOF prosthetic hand

## Abstract

In this research, the combination of fuzzy/PD and EMG signals, as direct command control, is proposed. Although fuzzy/PD strategy was used to control force position of the artificial hand, the combination of that with EMG signaling to voluntary direct command control is a novel method. In this paper, the EMG signal and its role in effective communication between a DC motor with a voltage trigger and neurofeedback are initially explained. Moreover, by introducing a filtration method, EMG pulses are obtained as stepping pulses with a signal-specific height of a voltage between 0 and 6 V, according to EMG domain voltage, with a time interval adapted from the EMG stimulus pulses. Two data points from each channel of EMG were extracted. The domain of the voltage of the EMG signal is impacted on the output of the fuzzy logic unit, and also the time amount between each stimulus of the EMG signal is the input of the PD controller. By this method, a user can influence grip position and grasping force of his/her prosthesis.

## Background

People who have lost a limb face many limitations in their life, so they expect that an alternative prosthetic can overcome those limitations and function like a living limb. However, this expectation has not yet fulfilled because of some sorts of shortcomings in technology [1].

Around the world, many groups of people suffer from amputation, caused by war, accidents, or diseases such as diabetes or mental disability.

The advancement of technology in each area of science has led to a remarkable evolution of available prosthetic devices, with functionality and appearance resembling living organisms more and more. The design of the prosthesis requires a multidisciplinary knowledge of biology, anatomy, electronics, mechanics, computer science, etc. The most important aspect of the design of prosthesis is the position of a disabled limb of the body and the amount of remaining muscle.

However, most of the research is conducted in a laboratory, and the issue is a loss of collaboration with the technology due to its multidisciplinary nature and the lack of adequate budget. There have been several samples of prosthetic hands, ranging from body-powered prosthesis to myoelectric hand, being manufactured and attempted in the commercial and for research. The choice of artificial hand is based on the necessity of the amputees. As usual, the prosthesis device actuator could be body-powered, pneumatic-powered, or electric-powered [2]. The body-powered artificial hand has the capability to actuate by muscles to direct the cable through a linkage. The benefits of body-powered prostheses are that they are cheaper and less expensive to fix. However, those prostheses are not cosmetically outward and are hard to operate with body power by some amputees. The electric-powered prosthetic hands that are battery-operated are utilized by the disabled due to their cosmetic appearance. However, these prostheses are costly. The disadvantage of battery-operated prostheses is extra weight and expense to maintenance. Although this happens a breakthrough in the operation of electric-powered prosthetic devices similarly, some disadvantages exist yet. These powered prosthetic hands must be operated by a pressure resistor, micro-switch, strain gauge, electromyogram signals (EMG), and electroencephalogram signals (EEG). There is a possibility of hybrid control method to improve the functionality of the devices.

Regardless of the operation of these prostheses, typically, the hand prosthesis is available with the mechanical design of prehensors, hooks, artificial hands, and a particular type of connection method depending on the user-specific bodily function.

The hook is a high-strength, lightweight, and low-cost device with unique gripping capability. The hook is constructed from metals such as aluminum, stainless steel, titanium, and chrome. Aluminum has less weight and lower intensity, and stainless steel has greater mass and strength. Titanium and chrome hooks have good power with less weight, but are comparatively more expensive. The hooks are not cosmetically intractable. They only are used for controlling body-powered devices. Prehensors are between hooks and artificial hands. Prosthetic prehensors are not cosmetically appealing and are in the same family as body-powered prostheses, such as prosthetic hooks. The unique types of connective devices are made to benefit amputees interested in recreational or sport activities.

**Fig. 1:**
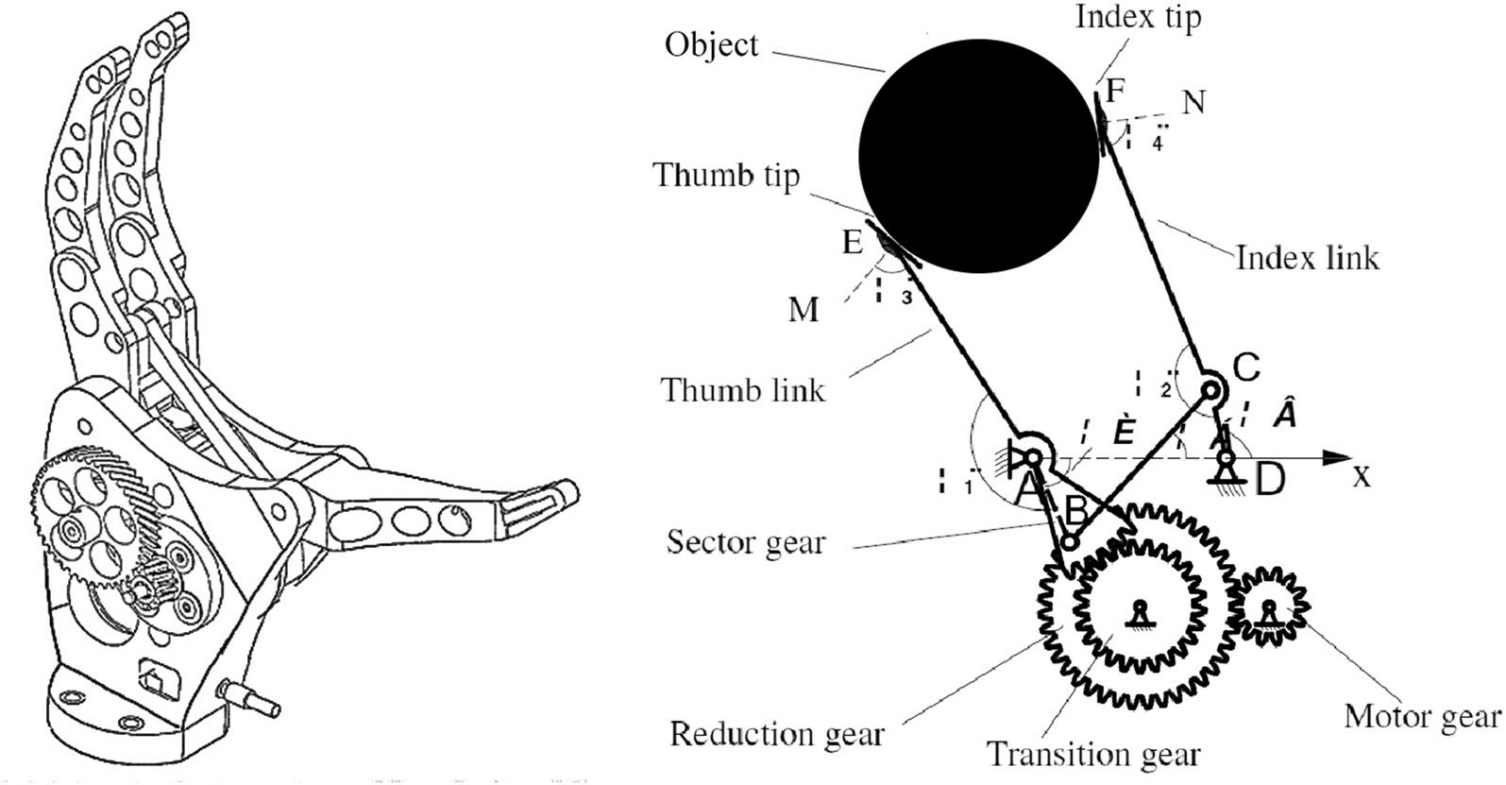
Exampled patent of the prosthetic hand structure [3]

**Fig. 2:**
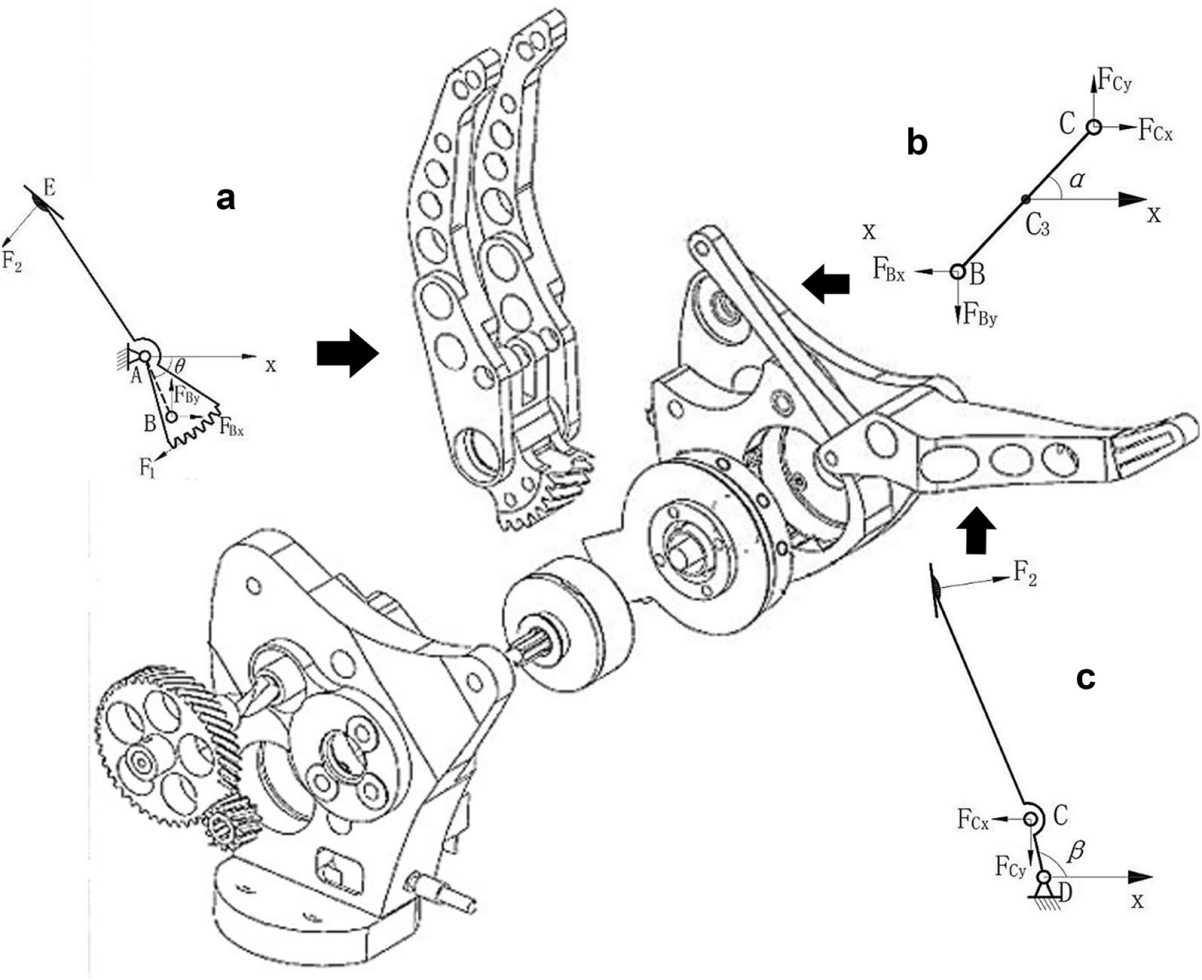
The mechanic’s analysis of links of the prosthetic hand, **a** thumb link, **b** connecting rod, **c** index link

Artificial hands are cosmetically agreeable but functionally substandard to hooks and prehensors. These prostheses may be controlled by using EMG signals, indicating the intention of the user. Prototypes are being created to control the hand robustly through the neural interface and through restoring the function of the nerves in the arm with targeted muscle reinnervation (TMR) surgery to move the hand. The current novel method is to control the prosthetic hand using EMG signals with various control schemes to interpret the muscle signals. Figures 1 and 2 shows the structure of an example patent of a patent of a prosthetic hand with two DOF [3].

### Some examples of amputee numbers

According to our research, the number of limb disabilities in China was up to 24.72 million people in 2010 [4]. Additionally, about 5320 physically disabled persons have held out in Shanghai since 2015 [5].

Besides, only 2.7 million Iranian individuals are disabled, with disability prevalence around 13 per 10,000 people in Iran [6]. The incidence of physical disability is greater than that of others. Disability prevalence is greater among men and increases at older ages in Iran [6]. In 2012 in the USA, more than 19.9 million people have a disability for lifting and grasping. This disability includes, for example, trouble lifting an object like a briefcase, or gripping a glass or a pencil [7], and there are additional estimated 1.9 million people living with amputations, and they currently live there [8].

These numbers have been challenged by disability organizations, claiming that families might have “ignored,” “denied,” or even “hidden” their disabled members during the census [9].

It is apparent that all amputees or disabled people need rehabilitation methods for improving his/her situation, so governments are engaging in this procedure to resolve this matter. People with amputations or disabilities want to improve their roles in society, but they don’t have jobs and usually assume the role of a consumer in their lifetime. Hence, our goal of designing of this control system is to alleviate the situation for people with amputations and disabilities to assist them in society.

It is possible for people with disabilities to be a producer in society and maintain a career, just as any other person. Amputees expect their prosthesis to have high functionality as if it is a living organism part of their physical structure. To achieve this, the researcher has presented a different type of control scheme for a prosthetic hand. Nevertheless, the prosthesis’s proficiency is still far from that of the living human organism. The design and control of these prostheses have a restriction in regard to application [1]. In the meantime, the development of the prosthetic hand and the micro-DC motors has replaced the mechanical cable; like the cable of break bicycle, the mechanical prosthetic hand initially had one degree of freedom. However, with the development of the scientific discipline, several degrees of freedoms (DOF) were extended with EEG/EMG signals to move the desired fingers [10].

### Recognition of EMG signal

The body-powered artificial hands are not mimicking the actual human hand gestures. The intention is to control a component which mimics the natural action. The other purpose of the control of hand may be exacted to control biosignal channels, which are received by the electrode. The electrode technology connects the biosignal to the prostheses. The user voluntarily controls the advanced prosthetic hand. This prosthesis is benefited some surface electrodes to connect the artificial hand by myoelectric signals to a user.

The sEMG signals to control the prosthesis are acquired from the surface of the skin and are favored due to their simplicity of admission. Additionally, this method is noninvasive. The ability of this kind of prostheses is less in surface EMG due to the limitation of recognizing the positions to acquire the signals. For the application of the surface electrodes, it is possible to define three to four potential locations from the remaining limb to receive signals for real-time control. However, getting the intramuscular EMG signals [11–13] is an invasive manner and requires the surgical ability for using the implantable myoelectric electrode. But the intramuscular EMG signals can provide access to the acquisition of the EMG signals from multiple muscles to add more degrees of freedom to control a prosthetic hand. It might be desirable to gain synchronous control of the prosthetic hand with the intramuscular EMG signals applying an implantable sensor.

Recently, the surgical operation TMR [14] has been used to distribute the nerves to different muscle sets, which can be measured from the surface to control the prosthetic hand. The use of TMR is efficient for training humeral amputees, and this method provides access for utilizing user intention.

### Myoelectric control designs

The EMG signal has been employed in hand prosthesis control since 1948 [15, 16]. Manufacturing commercial myoelectric hand prosthesis was started in 1957 at the Central Prosthetic Research Institute of Moscow to drive a stepper motor [17]. This issue was promoted to stationary electromagnet DC motor and electromagnetic relays. Next, the biocontrol approach had been extensively investigated, and a simple direct command control method was carried out. The focus of this paper is this subject. In the myoelectric control method, the voltage domain of EMG is applied to estimate the stage of the threshold of the received sEMG signals to fix on/off state and to determine the amount of the speed of the actuator. The command to move the prosthetic device is set by analyzing the amplitude measured by applying the origin assess square or indicating absolute value with the preset threshold. A broad mixture of control strategies have been developed to translate the information in the EMG and are typically categorized based on the nature of control as real-time control, robust control, and parallel control. Most of the control manner employed in prosthetic hand is of constant monitoring, and research is now being linked to the engaging control of the prosthetic hands. In subsequent control systems, the EMG signals are transformed by using the following methods:

1. On–off command control
2. Regression control
3. Direct command control
4. Extract and recognize pattern
5. Finite state machine control
6. Posture control
7. Proportional control

### Direct command control

The conventional direct command control is appropriate for two DOF. The prosthetic hands are controlled at a voluntary velocity in counterclockwise and clockwise orientations with full details. There are various control methods for direct command control. The simplest on–off command is based on a threshold of a potential domain of EMG signal to select the direction of control of the prosthetic hand. In this control manner, the prosthetic hand is moving at a fixed speed, independent of the rate of muscle contraction. The contemporary act control is probable with motors turned on and off and run at a constant speed [18]. In fact, the finite state machine control method is predefined as states and shifts among categories, and also it is predefined or decoded from the inputs [19, 20]. This method is proper for an indeterminate number of postures and may not be ideal for multifunctionality. Moreover, the mood transformation occurs from the EMG command until the aspired posture/function is preferred. These limitations can be alleviated by applying the pattern recognition procedure.

In 1981, Ray Barrett and Craig offered standard hybrid control method (position force). This technique successfully created adaptive control, where force control and position were designed on two separate tracks [21]. The control organization has two sets of loops. The primary control loop is applied on the interface between the prosthesis and the user in such a way that the electromyography control signal, coming from the skin surface where the muscle in question is located, is received in a noninvasive way by surface electrodes [22].

When an artificial hand grasps an object, it usually creates two conditions: before gripping the purpose and in a stage after grasping the object. In the first step, the hand has no contact with any objects in question, and the distance between the fingers and the purpose is unknown. Persons with disabilities expect prostheses to act as quickly as a natural hand.

When the hand almost reaches the object, it must touch it gently to avoid a sudden hit. In this stage [23, 24] the object’s mass is unknown. Without applying enough force to the prosthetic hands to the object, it may slip; on the contrary, the excessive force may result in damage to delicate objects, so to tackle these problems, prostheses need an efficient and fast method [25]. Hybrid control strategy used in the following prostheses has made a promising future possible [25–27].

In this paper, to realize the precise force control and almost no unwanted overshoot, a combined force control position is proposed. Control system provided for a prosthetic with two degrees of freedom and use of an EMG input to control operation not only controls the position and speed of the fingers and thumb before touching the object, but it also manages the force applied to the object after retrieving it [28].

Fuzzy hybrid controller/PD (force position), along with voluntary signal EMG, has an outside force feedback loop that minimizes the error between the actual forces using relative derivative control (PD) and an internal position feedback loop using the fuzzy controller (FLC) [28, 29] described below. An artificial dynamic model consists of a four-link system along with a DC micro-motor, two-wheel gear, and a virtual spring (as grasped things). Nowadays, the majority of commercial prosthetics are controlling. Often, when one channel is used, it means that the EMG signal is used as a switch (on/off) or (open/close). If two or more channels of EMG signals are used, this means that this prosthesis uses extraction pattern of signals for controlling.

## Methods

EMG is obtained from the skin surface, and it results from the muscle contractions (act/rest) muscle nerve cells [30]. In the late 1970s, the EMG signals were modeled as amplitude modulated by Gaussian noise in which variance was related to the force developed by the muscle contraction. Consequently, most commercial myoelectric modules are applied to control prostheses and are now based merely on one dimension of the EMG signals with the variance or mean positive value [31].

The SUVA hand was presented by Otto Bock. The grasp speed and grasp force were controlled by the intensity of the muscle signal. Two independent measurements and control systems ensure that the hand switches to grip force mode when an object is grasped, and the grip force is proportional to the muscle signal [32]. Touch Bionics Company has also introduced the external control loop generating an output signal, similar to a step signal, and then the dynamic equations of the prosthesis and internal control loop of the PD/fuzzy is being reviewed and discussed. Finally, the results of the simulation and control system are discussed.

A prosthetic hand called I-limb quantum is benefited from the EMG signal to move its fingers [33]. Nowadays, the majority of commercial prosthetic hands have one or two channels of EMG signal for controlling. Often, when one channel is used, it means that the EMG signal is used as a switch on/off or open/close.

**Fig. 3:**
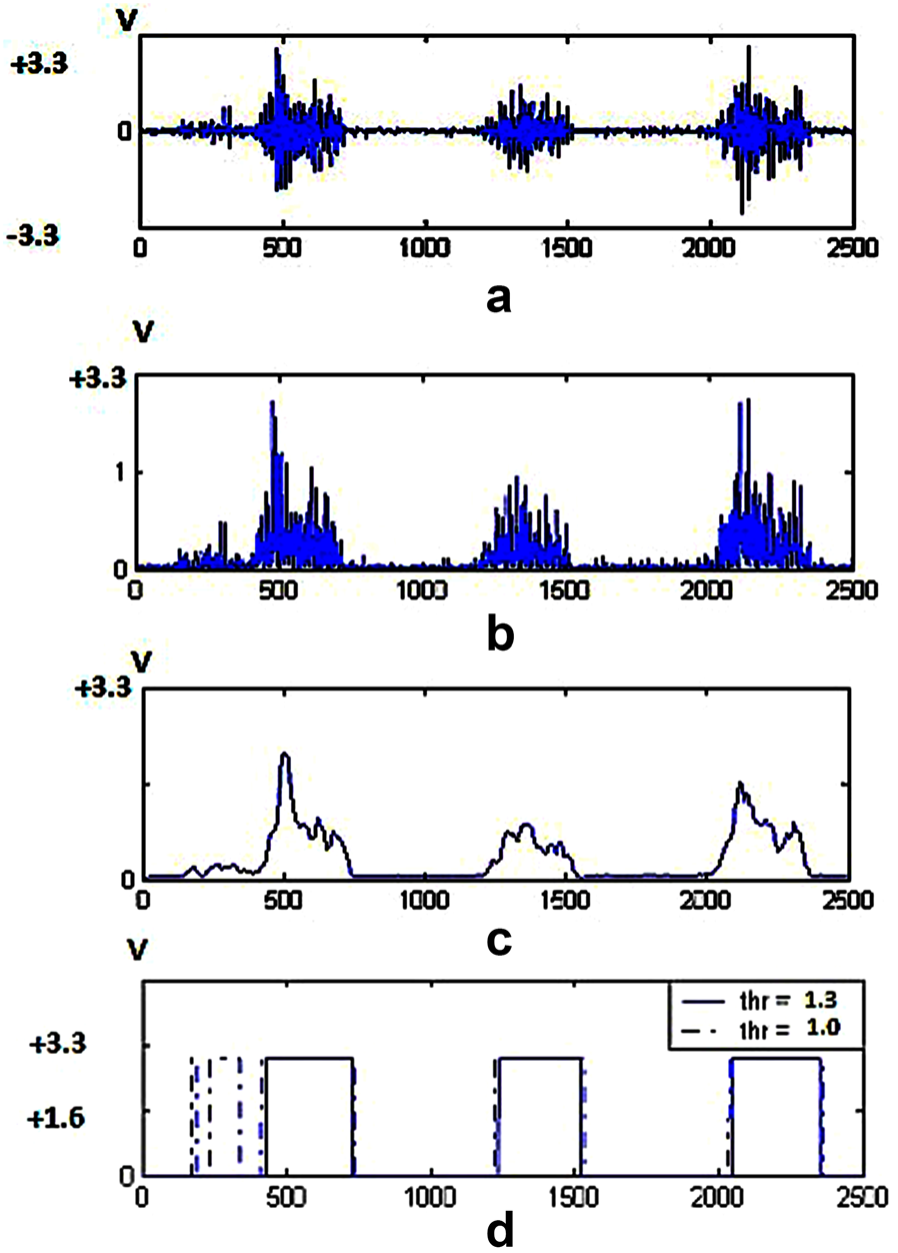
**a** Amplified EMG signal, **b** rectified EMG signal, **c** EMG signal after LFP, **d** output signal in external loop—the input signal of the internal control loop

The use of two or more channels of EMG signals implies that this prosthesis uses an extraction pattern of signals for controlling, as noted in [34–36]. The EMG signal can be generated from muscles. In this way, the EMG signals are received through stick electrodes. To extract the EMG, two electrodes placed on the muscle biceps or triceps brachial were used, and the third should be placed on the bone. The extracted data for sweating hands and feet control the velocity or force modes to suit the strong-willed users [30, 33]. Several authors successfully contributed to refining variance estimation of the myoelectric signal, for example, by applying a whitening filter or changing the smoothing window length to increase the number of states available from the surface EMG signal [37–39]. These techniques require a different muscle contraction for each controlled function, making the control of two or more joints very difficult. Because the effects of unwanted movement of the surrounding muscles create the additive noise, the EMG signal is dependent on physical condition of disabilities due to its natural characteristics. Only one input signal is used in the control system to decrease the impact of noises. The external control system with a single input can produce control command (speed and torque, clockwise and counterclockwise action). Speed and torque are obtained by the voltage domain of the EMG signal. So one of our parameters is the amount of the EMG voltage domain, and it was determined by putting two thresholds (up/down); the clockwise and counterclockwise actions were assigned. The single input senses EMG signal by electrodes on the skin surface of biceps brachial muscle. In the first phase signal is generated due to low voltage (about 4 mV) into the differential operational amplifier which amplifies voltage range (about 3.3 V) (Fig. 3).

**Fig. 4:**
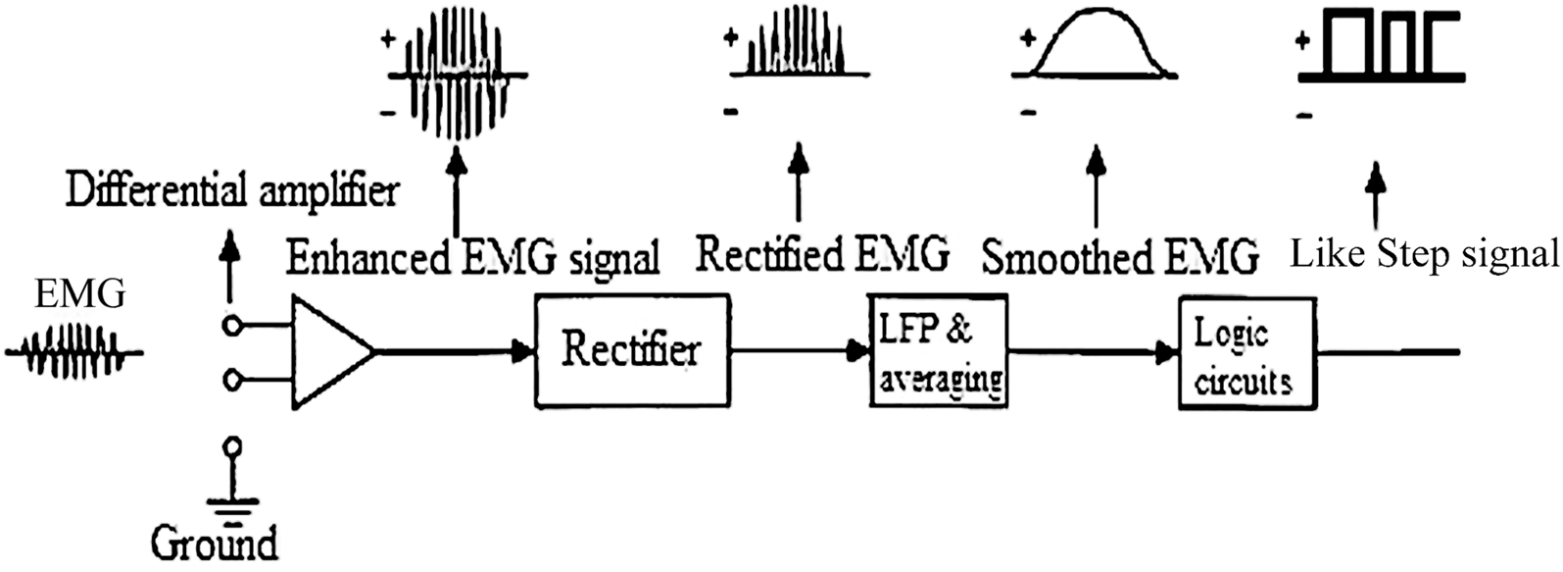
Overview of external control system

**Fig. 5:**
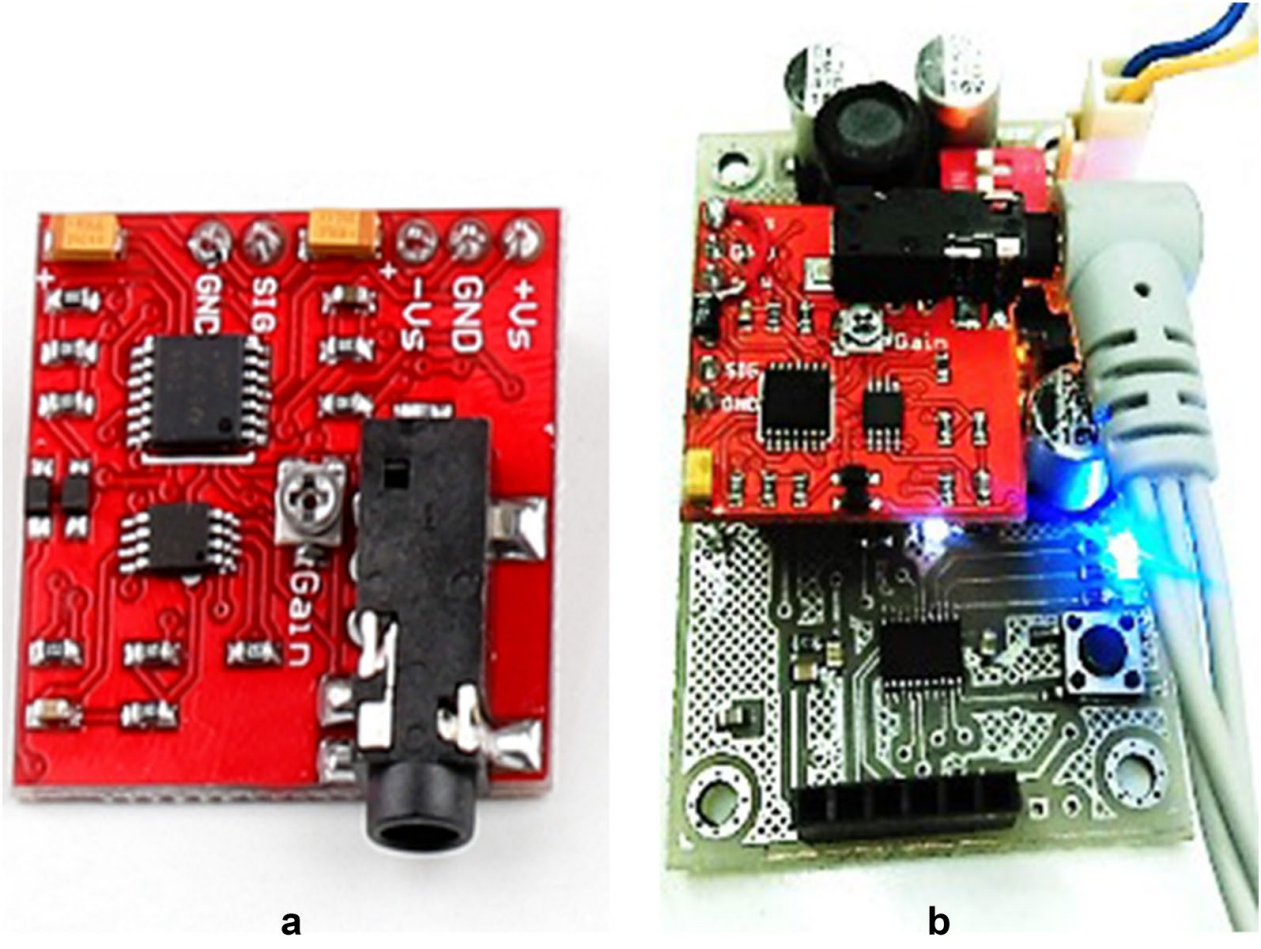
**a** Advantage technology EMG module, **b** EMG sender/receiver data

**Fig. 6:**
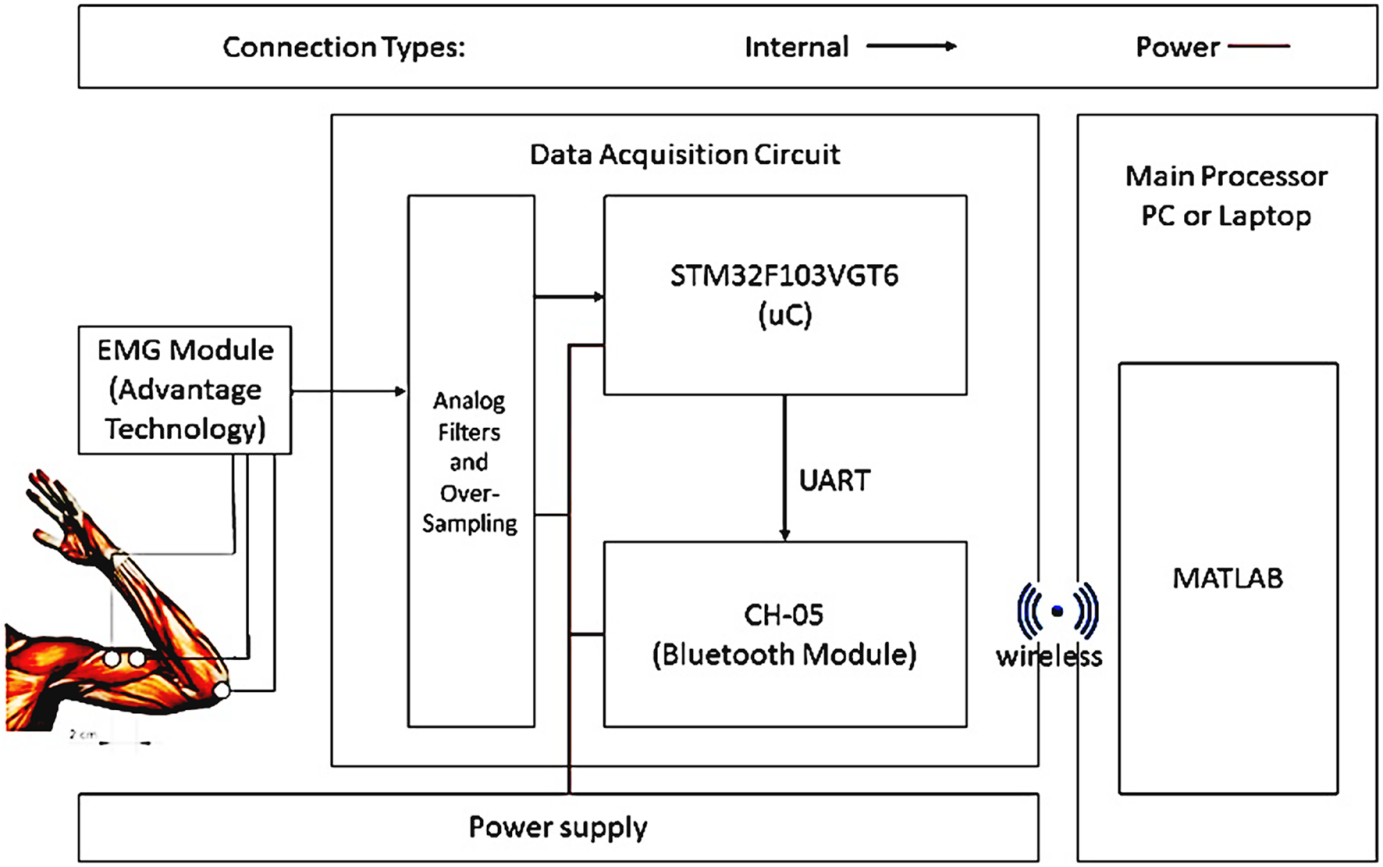
Schematic of transmitting EMG signal to MATLAB application

The next stage removes a part of noise range, Fig. 3a, and then acts on it to apply rectification, Fig. 3b, the EMG after passing through a low-pass filter, Fig. 3c, transfers to A/D of the microcontroller. The sampling rate of A/D is 1 μs. The output signal is similar to a step signal transmitted to the inner control loop. However, the difference is that the time delay of the muscle contraction (voluntarily action) determines the position of the fingers, and amplitude of the voltage of the pseudo-step signal determines the speed and the torque of the actuator. For clockwise and counterclockwise actions, two thresholds are identified as high and low (defined, it depends on each person). The internal control loop in standard mode puts the prosthetic hands in the open position. So the inner control loop after stimulation will change the finger position in the two states (clockwise/counterclockwise) as shown in Fig. 3d. The final design of external loop of the control system is shown in Fig. 4. To communicate with MATLAB, three steps were designed. The first step is to obtain the EMG signals via EMG sensor module for Arduino (advance technology) as shown in Fig. 5, and the output of this module is amplitude and filtered signal in analog; the second step is obtaining this signal by ADC. 12-Bit ADC is employed as the input port, and the type of ADC is SAR. By the oversampling conducted by the processor, the amount of noise is reduced and the number of useful bits’ increases. This ADC is in the STM32F407VGT microcontroller. The microcontroller, after applying the filter and oversampling of data, then transmits them via the UART connection to the CH-05 module (Bluetooth module). Next, the Bluetooth module pairs to the PC Bluetooth connection; in real time, MATLAB application can receive EMG data by serial communication functions. The schematic of this procedure is presented in Fig. 6. Each prosthesis is designed and used for specific purposes, regardless of its degree of freedom.

To introduce the mechanical section, in Figs. 1 and 2, the main structure of the prosthesis is presented, including one DC micro-motor, a linkage system, and a gear train. The thumb link and the sector gear are joined together through a hinge shaft. The micro-motor drives the sector gear through a reduction gear and a transition gear. The thumb link and the sector gear are linked into a whole through a hinge shaft. The sector gear runs the index finger through a connecting rod (BC). Therefore, the close and open function is realized. The point rays AD and A are settled as the origin and the *x*-axis of the Cartesian coordinate system, respectively. DC micro-motors with the metal gearbox are faced here Coulomb friction torque, which contains friction, and static friction has been known to have taken a constant angular velocity in the opposite direction to the motor. The dynamic model of the DC motor is given in Eq. 1:

1 $$\begin{aligned} J_{m} \dot{\omega } & = K_{\text{t}} i - B\omega - T_{\text{L}} - \text{sgn} (\omega )T_{\text{f}} \\ u & = L_{a} \dot{I} + K_{e} \omega + R_{a} i \\ \end{aligned}$$

where sign(*ω*) (rotor speed) is 0, − 1, or + 1, depending on EMG voltage domain, if the voltage increases the constant value is positive (in the counterclockwise action mode). And when the voltage decreases, the constant is negative (in the clockwise action mode) and a stable voltage amount of *ω* is 0. The angular velocity of the thumb’s link and the acceleration of it according to gear transmission properties (in Fig. 1) are given by:

2 $$\dot{\theta } = \omega /\left( {i_{1} i_{2} } \right), \;\ddot{\theta } = \dot{\omega }/\left( {i_{1} i_{2} } \right)$$

*J*_m_ is rotor inertia, *K*_t_ is tongue constant, is the current of the armature circuit, and is the viscosity coefficient of the motor. is rotor speed, *T*_L_ is load torque on the motor, _f_ is the friction torque of the motor, is the input voltage of the motor (EMG domain voltage plus **U**_f_),* L*_*a*_ is rotor inductance and the value of this is 4.5 × 10^−5^, $\dot{I}$ is derivative of ,* K*_*e*_ is back EMF constant at 6.92 × 10^−3^, and* Ra* is the terminal resistance of the motor.

According to the geometric properties of a four-link mechanism, the equivalent equations only are listed in Eq. 3:

3 $$\begin{aligned} & L_{2} \cos \theta + L_{3} \cos \alpha = L_{1} + L_{4} \cos \beta \\ & L_{2} \sin \theta + L_{3} \sin \alpha = L_{4} \sin \beta \\ \end{aligned}$$

*L*_1_ is the length of the link 0.0402 (m), *L*_2_ is 0.0190 (m), *L*_3_ is 0.0430 (m), *L*_4_ is 0.0143 (m), is angled from *x*-axis to BC, and is angled from *x*-axis to CD. Under the rules of classical Newtonian mechanics, balance equations obtained are given in Eq. 4 as follows:

4 $$\begin{aligned} & J_A\ddot{\theta } = F_{1} R_{0} - F_{2} L_{3} \sin \varTheta_{3} - F_{\text{Bx}} L_{2} \sin ( - \theta ) - F_{\text{By}} L_{2} \cos \theta \\ & J_{\text{C3}} \ddot{\alpha } = F_{\text{Bx}} L_{c3} \sin \alpha + F_{\text{Cx}} (L_{3} - L_{\text{C3}})\,\text{Sin}\, \alpha - F_{\text{By}} L_{\text{C3}} \cos \alpha \\ & - \;F_{\text{Cy}} \left( {L_{3} - L_{\text{C3}} } \right)\cos \alpha \\ & J_{D} \ddot{\beta } = F_{\text{Cx}} L_{4} \sin \beta + F_{\text{Cx}} L_{4} \cos (\pi - \beta ) - F_{2} \sin \varTheta_{4} \left[ {L_{6} + L_{4} \sin (\varTheta_{2} - \pi /2)} \right] \\ \end{aligned}$$

*J*_*A*_ is inertia of thumb link to *A*, _0_ (radius of sector gear) is 0.0252 (m), and $\theta$ is − 1.51  to − 0.88 rad. Θ_3_ is 1.27 rad, ¨ is derivative of , Θ_2_ is 2.97 rad, _6_ is 0.0600 (m),* J*_*D*_ is an inertia index link to *D*, Θ_4_ is 1.27 rad that the *F*_1_ force resists the gear shift thumb. The *F*_Bx_, *F*_By_, *F*_Cx_ and *F*_Cy_ forces acting on the interface bar that depends entirely on *F*_1_, *F*_2_ force on the fingertips are perpendicular to the surface fingertips *L*_C3_.

The distance is identified from point B and point *C*_3_. *C*_3_ is the center of the mass to link BC. The force on the link BC is obtained from Eq. 5:

5 $$\begin{aligned} m_{3} a_{\text{C3x}} & = F_{\text{Cx}} - F_{\text{Bx}} \\ m_{3} a_{\text{C3y}} & = F_{\text{Cy}} - F_{\text{By}} \\ \end{aligned}$$

*m*_3_ is the mass of BC, based on the gear shift characteristics, and torque force on the DC motor is equal to Eq. 6:

6 $$T_{L} = F_{1} R_{0} /(i_{1} i_{2} )$$

*i*_1_ (ratio between motor gear and reduction gear) is 10:1. And *i*_2_ (the ratio between transition gear and sector gear) is 12.5:1, which defines the coordinates of the tip of the thumb and the tip of the index finger, respectively, *E* (*X*_e_, *Y*_e_) and *F* (*X*_f_, *Y*_f_). The dynamics model of the prosthesis system, composed of the motor mathematical model and the four-link system, is built by Eqs. 1, 2, 6, 7, which yields the equation:

7 $$f(\theta ) \cdot \ddot{\theta } + g(\theta ) \cdot \theta^{2} = F_{1} \cdot h_{1} (\theta ) + F_{2} \cdot h_{2} (\theta )$$

where *f*(*θ*) and *g*(*θ*) are very complex nonlinear functions of variable *θ*, respectively. The *h*_1_(*θ*) and *h*_2_(*θ*) are also nonlinear functions. Assigning state variables *x*_1_, *x*_2,_ and *x*_3_ corresponding to *i*, *θ* and $\dot{\theta }$, respectively, yields the following form of the system model:

8 $$\dot{x}_{1} = (u - K_{\text{e}} i_{1} i_{2} x_{3} - R_{\text{a}} x_{1} )/L_{\text{a}}$$

9 $$\dot{x}_{2} = x_{3}$$

10 $$\dot{x}_{3} = \frac{{J_{m} i_{1} i_{2} \left[ {K_{t} x_{1} - Bi_{i} i_{2} x_{3} - \text{sgn} (x_{3} )T_{f} } \right] - R_{0} g(x_{2} )x_{3}^{2} - F_{2} R_{0} h_{2} (x_{2} )}}{{J_{m} (i_{1} i_{2} )^{2} h_{1} (x_{2} ) + R_{0} f(x_{2} )}}$$

According to the geometric relationships between *E* and *F* fingertips as expressed in Eq. 11:

11 $$S = \left| {EF} \right| = \sqrt {\left( {x_{e} - x_{f} } \right)^{2} - \left( {y_{e} - y_{f} } \right)^{2} } = S\left( {x_{2} } \right)$$

**Fig. 7:**
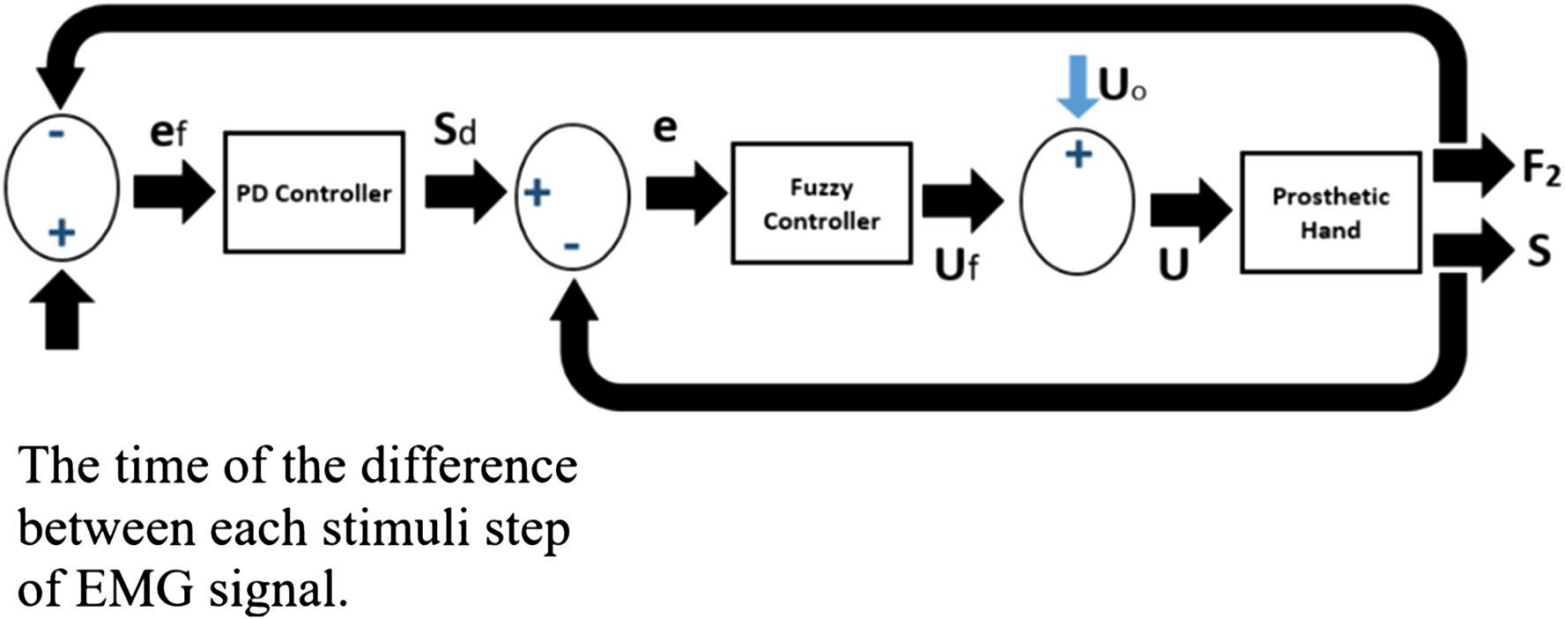
Full version of prosthesis control system. *U*_0_ is voltage domain of the filtered, rectification, and amplified EMG signal (between 0 and 6 V)

*s* is determined to be 0–0.12 (m), and fuzzy/PD hybrid controller with voluntary EMG signal to control (force position) is designed. It also has a double-loop feedback for the internal control system. The external loop of internal control system is force control, and the inner loop is to control the situation the outer loop and position. The PD controller is used in outer, because the PD controller consists of two parallel types of derivative and integral controllers. The derivative controller has quickly adapted to the input changes, so if the fast response is needed, this controller can be used. However, the derivative action enhances the input noise, so the derivative control is not used separately and needs to combine with another method. The other cause of applying the PD controller is the servomechanism characteristics of the mechanical/controlling system. This system contains a DC micro-motor, and two control loops have a direct or indirect effect on the position of the micro-motor (the position is equal to velocity integral, and speed is the rate of modifying of position) [18] (Fig. 7)

12 $$S_{\text{d}} = K_{\text{Fp}} e_{\text{F}} + K_{\text{Fd}} \dot{e}_{\text{F}}$$

*e*_F_ is the error of the force and amount of the time of the difference between each stimuli step of EMG signal, *S*_d_ optimal distance, *K*_FP_ the coefficient of proportionality, and *K*_Fd_ derived factor *S*_d_. The PD controller output determines the optimal distance between the tip of the thumb and index links [40].

The internal control loop, fuzzy logic controller (FLC), is applied to improve anti-interference performance and adaptation of system parameters applied by the fuzzy controller inputs in the position error; position error “*e*” is defined by Eq. 13:

13 $$e = S_{\text{d}} - S$$

**Fig. 8:**
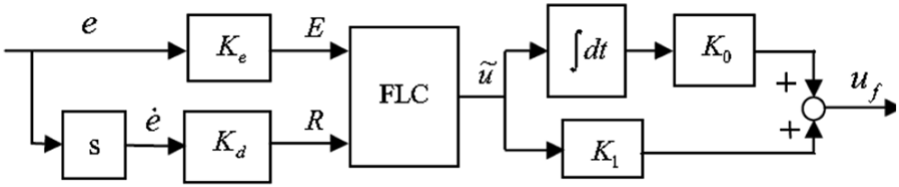
Structure of fuzzy logic controller

**Fig. 9:**
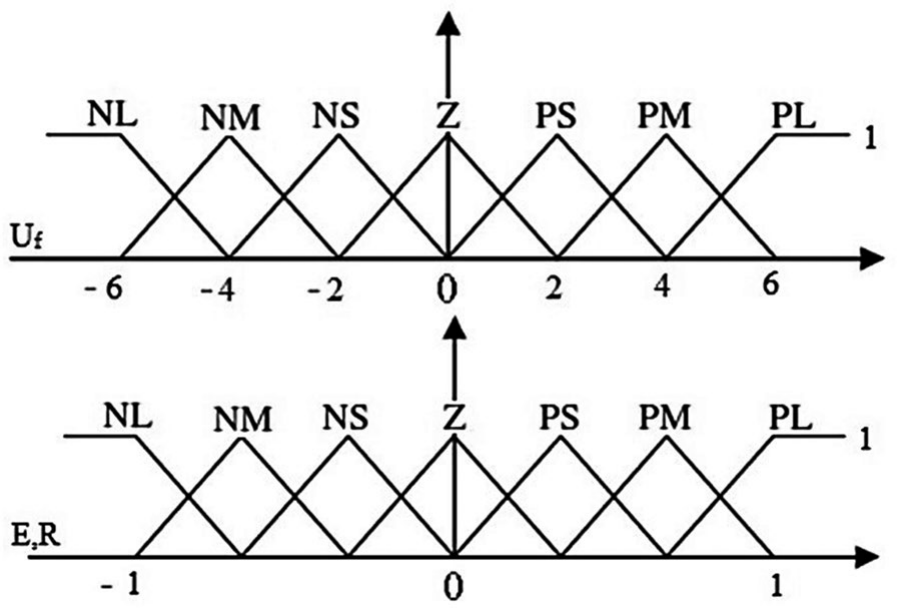
Triangular membership functions for input and output

Here *S* is the actual distance between the index and thumb finger. Extra details of the fuzzy controller design are available at [28, 29, 41, 42]. In this article, the overall structure of a fuzzy control system for controlling is considered in Figs. 8 and 9. First, input gets the derivative of the distance error. And the other input obtains the real distance error. The values of each entry have clustered into seven states, and each interval is labeled, see Table 1.

**Table 1:** Rules of fuzzy controller

R/E	NL	NM	NS	Z	PS	PM	PL
PL	Z	PS	PM	PL	PL	PL	PL
PM	NS	Z	PS	PM	PL	PL	PL
PS	NM	NS	Z	PS	PM	PL	PL
ZR	NL	NM	NS	Z	PS	PM	PL
NS	NL	NL	NM	NS	Z	PS	PM
NM	NL	NL	NL	NM	NS	Z	PS
NL	NL	NL	NL	NL	NM	NS	Z

### Fuzzy uncertain structure controller [43]

The FUSC, fuzzy uncertain structure controller, method is presented to understand the control for uncertain system with noise. First, the following sliding level is introduced in Eq. 14:

14 $$S\left( t \right) = C^{T} E\left( t \right) = e_{n} \left( t \right) + \mathop \sum \limits_{i = 1}^{n - 1} c_{i} e_{i} (t) \quad i \le {\mathcal{N}}$$

where *C* = [*c*_1_, *c*_2_…, *c*_*n*−1_, 1]^T^ is selected such that the distribution of the roots of characteristic equation *p*^*n*−1^_+_ *c*_*n*−1_ *p*^*n*−2^_+_ ··· _+_ *c*_2_*p*_+_ *c*_1_ = 0 is on the left side of the complex plane to make the following system stable Eq. 15:

15 $$e_{n} \left( t \right) + \mathop \sum \limits_{i = 1}^{n - 1} c_{i} e_{i} \left( t \right) = 0$$

Then Eq. 16:

16 $$\begin{aligned} & \dot{s}\left( t \right) = \dot{e}\left( t \right) = e_{n} \left( t \right) + \mathop \sum \limits_{i = 1}^{n - 1} c_{i} e_{i + 1} \left( t \right) \\ & = f\left( x \right) - x_{rn} \left( t \right) + d\left( t \right) + b\left( t \right).u\left( t \right) + \mathop \sum \limits_{i = 1}^{n - 1} c_{i} e_{i} \left( t \right) \\ \end{aligned}$$

Based on Lyaponov plane and VSC theory, the following analytical result can be achieved. Initially, a fuzzy auxiliary controller *D*(*t*) is built to determine the control noise *d*(*t*). The fuzzy rules are given by:

$$\begin{array}{*{20}l} {{\text{IF}}\;{\text{Sd}}\left( {\text{t}} \right) > 0\;{\text{THEN}}\;{\text{D}}\left( {\text{t}} \right)\;{\text{should}}\;{\text{be}}\;{\text{enhanced,}}} \hfill \\ {{\text{IF}}\;{\text{S}}_{\text{d}} \left( {\text{t}} \right) < 0\;{\text{THEN}}\;{\text{D}}\left( {\text{t}} \right)\;{\text{should}}\;{\text{be}}\;{\text{reduced,}}} \hfill \\ \end{array}$$

17 $$S_{\text{d}} (t) = \dot{s}\left( t \right) + \xi .\text{sgn} \left( {s\left( t \right)} \right)$$

The term under consideration *D*(*t*) can assume a higher value. If it is too large, this would lead to some intense control problem in practice. Therefore, based on the integration method, the small value Δ*D*(*t*) is considered to replace by *D*(*t*) for their relations in Eq. 18:

18 $$D\left( t \right) = G\mathop \int \limits_{0}^{t} \Delta D\left( s \right){\text{d}}s$$

*G* is the coefficient of proportionality. Sd indicates the fuzzy input *S*_d_(*t*), and Δ*D* defines the fuzzy output Δ*D*(*t*). The fuzzy sets the input and the output are defined, respectively, as:

19 $$\begin{aligned} S_{\text{d}} = \left\{ {{\text{NL}},\;{\text{NM}},\;{\text{NS}},\;{\text{Z}},\;{\text{PS}},\;{\text{PM}},\;{\text{PL}}} \right\}, \hfill \\ \Delta D = \{ {\text{NL}},\;{\text{NM}},\;{\text{NS}},\;{\text{Z}},\;{\text{PS}},\;{\text{PM}},\;{\text{PL}}\} , \hfill \\ \end{aligned}$$

The clustering and range values for each input to seven items are tagged with small negative (SN), medium negative (MN), large negative (LN), zero (Z), small positive (SP), moderately positive (MP) and large positive (LP). The rules of the Fuzzy control are adopted in the form in Eq. 20:

20 $${\text{If}}\;\left( {{\text{R}}\;{\text{is}}\;{\text{PL}}} \right)\;{\text{and}}\;\left( {{\text{E}}\;{\text{is}}\;{\text{NL}}} \right)\;{\text{then}}\;\left( {{\mathbf{U}}_{\text{f}} \;{\text{is}}\;{\text{Z}}} \right)$$

Derived errors and input errors membership degree between (− 1, 1) and output membership degree between (− 6 and 6) are located. Because the fuzzy controller output is directly imported to the input voltage DC micro-motors, it is fitted with a rated voltage. Therefore, it is fitted with a rated voltage. To implement simulation systems, linear objects will be considered as virtual springs. After denying a prosthesis is taken by the thumb of force in the opposite direction perpendicular to it, which is named *F*_2_. Fix the constant spring factor we consider.

21 $$k = 200\;{\text{N/m}},\;0 \le \, l \le 12$$

*l* is the maximum distance the two fingers, and *k* is the constant of virtual spring, So* F*_2_ is equal to Eq. 22:

22 $$F_{2} = k \times \left( {S - S_{\text{o}} } \right),\quad 0 \le S \le S_{0}$$

For force control loop parameters (PD), the following values have been selected in Eq. 23:

23 $$K_{\text{Fp}} = 0.833464,\;K_{\text{Fd}} = 1.3$$

And position control loop parameters (fuzzy controller) have selected the following values in Eq. 24:

24 $$K_{0} = 1,\;K_{d} = 0.000002,\;K_{e} = 0.00006,\;K_{t} = 100$$

**Fig. 10:**
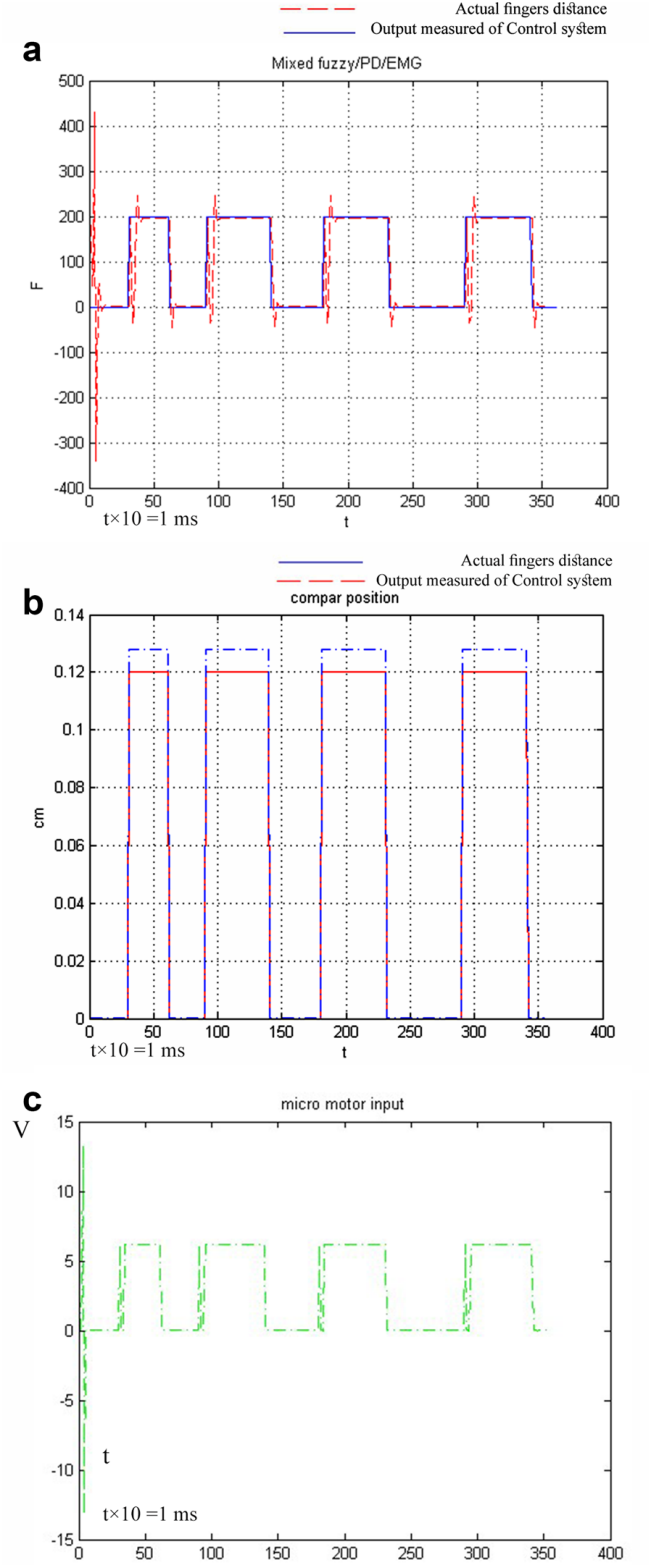
**a** Control system response of step signal (EMG). **b** Comparison of the real distance of two fingers with the calculated range by the control system. **c** The applied voltage from the control system to the actuator

The spring constant *k* = 200 N/m is also taken into account, and the results of the simulation in Fig. 10 show that the control scheme is proposed by combining fuzzy/PD (force position), along with the EMG prosthetic hand which is the excellent performance of the system. Note that outside forces are not too artificial and can accurately calculate the force of various objects with the user will be required when the virtual spring has little effect on the control signals. The DC motor has a dead-band because of Coulomb friction, viscosity and friction Backlash phenomenon that makes the force control problems at the initial moment very difficult. Because of the difficulty, the negative voltage to the motor controller is careful to keep the force grab, and the motor input control signal is applied repeatedly.

## Discussion and conclusion

In this paper, the EMG signal and its role in effective communication between a DC motor with voltage trigger and neurofeedback are explained initially. By introducing and then by introducing a filtering method, EMG pulses are obtained as stepping pulses with a signal-specific height of a voltage between 0 and 6 V, according to EMG domain voltage, with a time interval adapted from the EMG stimulus pulses. Thus, two data points are extracted from one EMG channel. Briefly, voltage domain of the EMG, that impact on output of FLU, and also time amount between each stimulus of EMG signal as the input of PD controller. By this method, a user can influence position and power gripping (Force = *F*_1_) of his prosthesis. The data produced by these filters will generate the stimulation voltage of the actuator of prosthetic hand system for the subject of this article. Using the relations of trigonometry and differential equations on time resulted in two-dimensional kinetics and dynamics of the motion of prosthetic hand mechanism (Eqs. 1–10); with the help of these relationships, the distance between the clamping device of the artificial limb and its linear velocity is influenced by the angle, and angular velocity of the main propulsion DC motor is achieved. The equations of a linear spring are used to simulate the force applied between the jaws of the prosthetic hand and displacement between them (Eqs. 11, 12, 13, 21). Then, fuzzy control and PD control system are used (Eqs. 11–24), the relationship between the displacement feedback, the speed and the force applied between the two jaws will be created with the rotational speed and position and torque of the actuator. A stable force as a step signal with a variable time interval was desired to test the control method. Figure 10a shows that system by acquiring EMG signal as input can be convergent in output with the desired force. The overshoot is generated as the initial value of the control system, and the overshoot repeated at each step is created by motion artifact noise, at the time of contraction of the muscle. Figure 10b, c, respectively, demonstrates and compares the real distance of two fingers with the calculated range from the control system and the applied voltage from the control system to the actuator. The repeated overshoots in Fig. 10c are caused by the motion artifact noise that impacted by *U*_0_ as an extra voltage to the DC motor.

Proper response and a significant follow-up between the two desired signals and the actual performance of the system can show the excellent functioning of the control system and the idea of utilizing the EMG signals and turning it on to the step pulse signals of the motor’s voltage. In the future, it will be applied that the control system is implemented practically on hand prosthesis with 5 degrees of freedom and examines the duration of adaptivity of a person with disability, the performance, and the response of the control system with the real environment.
